# Characterisation of trocar associated gas leaks during laparoscopic surgery

**DOI:** 10.1007/s00464-021-08807-1

**Published:** 2021-11-03

**Authors:** Daniel Robertson, Frank Sterke, Willem van Weteringen, Alberto Arezzo, Yoav Mintz, Felix Nickel, the Technology committee of the European Association for Endoscopic Surgery (EAES), Luigi Boni, Ludovica Baldari, Thomas Carus, Manish Chand, Hans Fuchs, Fanny Ficuciello, Stefania Marconi, George Mylonas, Young Woo Kim, Kiyokazu Nakajima, Marlies Schijven, Pietro Valdastri, Chen Sagiv, Pietro Mascagni, Piotr Myśliwiec, Wanda Petz, Francisco Sánchez-Margallo, Tim Horeman

**Affiliations:** 1 Department of Biomechanical Engineering, Faculty of Mechanical Engineering, Delft University of Technology, TU Delft, Mekelweg 2, 2628 CD Delft, The Netherlands; 2 Department of Paediatric Surgery, Erasmus MC Sophia Children’s Hospital, University Medical Center Rotterdam, Rotterdam, The Netherlands; 3 Department of Surgical Sciences, University of Torino, Torino, Italy; 4 Department of General Surgery, Hadassah Hebrew University Medical Center, Jerusalem, Israel; 5 Faculty of Medicine, Hebrew University of Jerusalem, Jerusalem, Israel; 6 Department of General, Visceral, and Transplantation Surgery, Heidelberg University Hospital, Im, Neuenheimer Feld 420, 69120 Heidelberg, Germany

**Keywords:** Surgical safety, Surgical smoke, Laparoscopic equipment, Gas leak, Carbon dioxide

## Abstract

**Background:**

During laparoscopy, the abdominal cavity is insufflated with carbon dioxide (CO_2_) that could become contaminated with viruses and surgical smoke. Medical staff is potentially exposed when this gas leaks into the operating room through the instruments and past trocar valves. No detailed studies currently exist that have quantified these leakage pathways. Therefore, the goal of this study was to quantify the gas leakages through trocars and instruments, during minimally invasive procedures.

**Methods:**

A model of the surgical environment was created, consisting of a rigid container with an interface for airtight clamping of laparoscopic equipment such as trocars and surgical instruments. The model was insufflated to 15 mm Hg using a pressure generator and a pneumotachograph measured the equipment gas leak. A protocol of several use cases was designed to simulate the motions and forces the surgeon exerts on the trocar during surgery.

**Results:**

Twenty-three individual trocars and twenty-six laparoscopic instruments were measured for leakage under the different conditions of the protocol. Trocar leakages varied between 0 L/min and more than 30 L/min, the instruments revealed a range of leakages between 0 L/min and 5.5 L/min. The results showed that leakage performance varied widely between trocars and instruments and that the performance and location of the valves influenced trocar leakage.

**Conclusions:**

We propose trocar redesigns to overcome specific causes of gas leaks. Moreover, an international testing standard for CO_2_ leakage for all new trocars and instruments is needed so surgical teams can avoid this potential health hazard when selecting new equipment.

In minimal access surgery, the surgical field is exposed by insufflation of pressurized carbon dioxide gas (CO_2_). Trocars provide access to the body cavity for both gas insufflation and insertion of a scope and instruments. In clinical practice, a perfect gas seal is difficult to achieve, with minor leaks of CO_2_ through the incision, the trocars and the surgical instruments. In certain procedures with higher pressures, longer operating times, or frequent instrument changes this can result in the leakage of several hundred litres of gas into the operating theatre [1].

One of the main concerns is the exposure of operating theatre personnel to surgical smoke and other aerosols. There have been studies measuring the composition of smoke in laparoscopy, in which carcinogenic compounds were found [2, 3]. It has been proven that peritoneal fluids can contain pathogens such as viral particles [4] that can be carried into the operating theatre through insufflation gas leakage. There have been rare documented cases where surgical smoke containing viruses like the human papilloma virus (HPV), have led to human transmission [5–8].

Covid-19 has revived the concerns over peritoneal gas leakage potentially containing harmful substances. Recently, a number of studies have been published on the safety of performing laparoscopic surgery on Covid-19 positive patients [9, 10]. Considering the current knowledge on the transmission and virulence of Covid-19 the spread through insufflation gases cannot be ruled out [11].

Cross-contaminations in the OR can be prevented by a number of different measures. These range from improved airflow to specific smoke evacuation devices. Although laminar airflow reduced the number of smoke particles near OR personnel, these systems cannot counteract a strong influx of contaminated gas [12]. Smoke evacuation devices aim to prevent particles escaping into the OR entirely. However, leakages through laparoscopic equipment could undercut both protective measures.

Thus far, one study has measured the flow of gas through a cannula and instrument. However, the contribution of either the cannula or instrument was not quantified [13, 14]. The use of different combinations of trocars and instruments will likely result in varying leakage performance. The choice of equipment might cause OR personnel to be exposed to contaminated gas. Therefore, this study aims to investigate gas leakage through representative and commonly used trocars and instruments. A model was developed to measure gas leak due to trocar-instrument interactions that occur during a laparoscopic procedure.

## Materials and methods

### Trocars and instruments

**Fig. 1:**
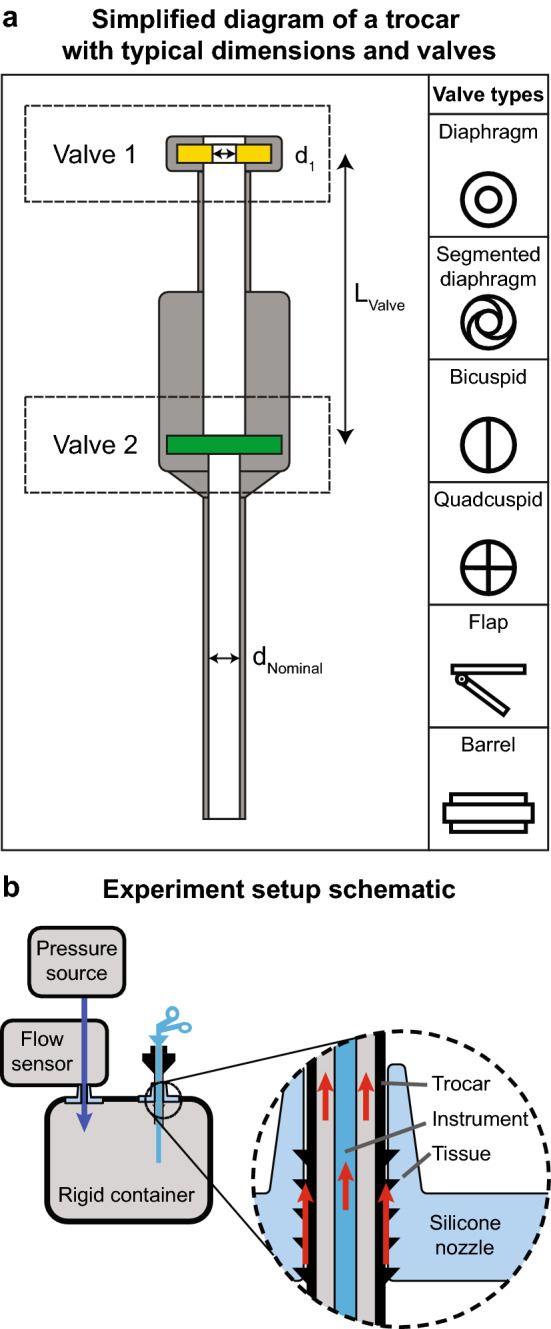
**a** Trocar dimensions and valve types, *d*_nom_ was used for categorization. The distances between the valves and the lumen diameters were measured. Six different types of valves could be distinguished: diaphragm, segmented diaphragm, flap, barrel, bicuspid and quadcuspid valve. **b** The leak measurement setup and leak pathways: A rigid container that was pressurized using an external pressure source. The flow needed to keep the rigid container pressurized was measured at the inlet, the inlet flow equalling the leak through the trocar and/or instrument. In an OR setting, CO_2_ can leak through three pathways: through the instrument, through the trocar and between the trocar and tissue. In this setup a silicone membrane was used to prevent leak through the tissue pathway

To quantify the problems related to trocar and instrument leakage during laparoscopy, surgeons from hospitals throughout Europe were asked to provide trocars and instruments that are used in their hospitals. As this study was a technical equipment evaluation, no IRB permission was required. All materials were checked for defects and categorized before testing. Only trocars with a nominal size of 5 mm and 12 mm were included, duplicate trocars were excluded. No scopes were included in the instrument measurements. The trocars and instruments were categorised based on size and reusability: reusable, disposable or reposable (partially reusable). Before performing the measurements, relevant trocar properties such as: the number of valves; valve type; valve lumen diameters and inter-valve distances (*L*_valve_) were noted, which are shown in Fig. 1a.

### Model

Three potential leak pathways were identified: (1) through the trocar; (2) through the instrument; (3) through the incision between the tissue and trocar. For this study, only pathways 1 and 2 were of interest. To avoid the third ‘tissue leak’ pathway, two custom nozzles were designed to provide an airtight seal for 5 and 12 mm trocars. These nozzles were made of silicone and had inner diameters that were smaller than the smallest outer diameter of a trocar. The shape of these nozzles is shown in Fig. 1b.The airtight seal was verified with a soap bubble test.

To investigate pathways 1 and 2, a rigid container was used as a model for trocar leak during a laparoscopic procedure. A schematic of the model is shown in Fig. 1b. The rate of gas leakage is mainly dependent on variables such as the intra-abdominal pressure and the resistance to gas flow of the trocar and instrument. In practice, gas leak and CO_2_ absorption cannot be distinguished from each other. Abdominal compliance can also affect the incision leak around the trocar. Therefore, this model isolates the leakage through trocars and instruments. The model was insufflated using an external pressure source which pressurized room air to 15 mm Hg to comply with standard intra-abdominal operating pressures.

### Protocol

The effects of trocar-instrument interaction were studied by performing a series of manipulations and an instrument insertion. The different tests represent conditions that could occur during surgery. These tests are designed to investigate performance aspects of the specific valves of the trocar. All trocars underwent baseline measurements, manipulations and an insertion test.

#### Baseline

During the baseline measurement, the trocar was empty, and valve 2 prevented gas from escaping. The instruments were measured when directly inserted into the silicone nozzle, without a trocar.

1. *Baseline*: Only empty trocar or individual instrument.

#### Manipulation

When manipulating tissue, the instrument is inserted into the cannula with the shaft protruding all valves. During manipulation, valve 1 creates a seal around the shaft of the instrument, while valve 2 is kept open by the instrument.

**Fig. 2:**
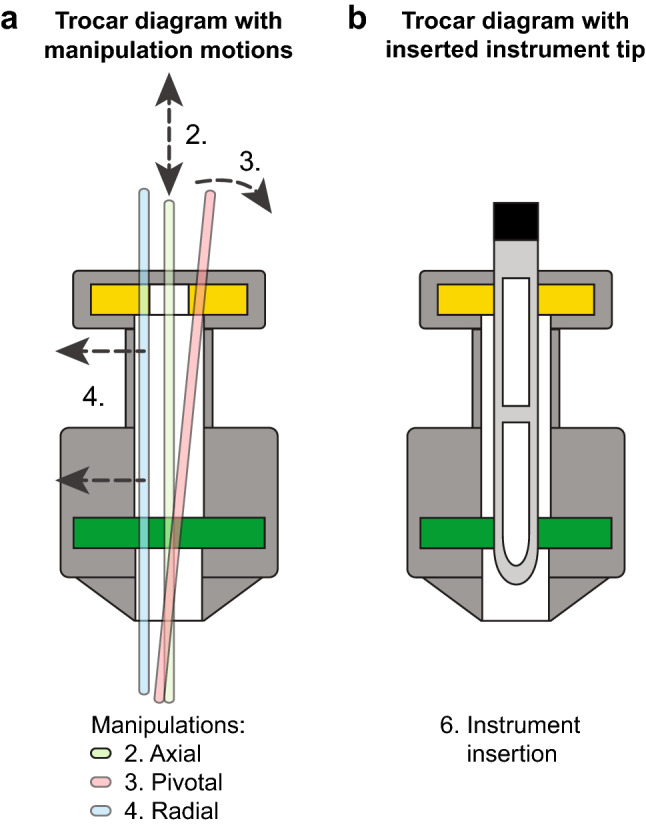
**a** Manipulations of the rod within the trocar, in axial (2), pivotal (3) and radial (4) directions. Axial manipulation oscillation are performed with a 5 cm amplitude. During the pivotal and radial manipulation the rod is maximally displaced. **b** During the instrument insertion test, a grasper with a fenestrated structure was inserted into the trocar and kept in contact with both valves to allow for an open passage of gas

Manipulations on all trocars were performed manually with two solid steel rods to mimic the use of a surgical instrument. Using a solid rod, the leakage through the trocar was isolated. The rods used had a diameter of 5 ± 0.02 mm and 12 ± 0.02 mm for the respective size of trocar and a length of 350 mm, providing sufficient length to manipulate the rods on both ends protruding from the trocar. The 12 mm trocars with diaphragms that were smaller than 5 mm were tested with the 5 mm and 12 mm tools. Four different manipulations were manually performed as shown in Fig. 2a. The manipulations were:

(2) *No manipulation*: the rod placed through all valves in the trocar and held in an upright position by an instrument holding arm by which no forces or displacements were exerted on the rod or trocar.
(3) *Axial manipulation*: five oscillations of the rod with a 5 cm amplitude axial to the trocar.
(4) *Pivotal manipulation*: pivoted placement of the rod within the trocar until the maximum angle allowed by the trocar entry.
(5) *Radial manipulation*: the rod moved within the trocar parallel to the trocar axis until maximum displacement.

#### Insertion

During instrument insertion, both valves determine the leakage performance. When a fenestrated instrument tip is longer than the distance between two valves, it opens both valves simultaneously and could allow leak through the instrument tip as seen in Fig. 2b. The insertion test was performed with a 5 mm fenestrated atraumatic grasper with a 4.9 mm diameter and a 28 mm tip length, and with a 12 mm stapler with a 12.2 mm diameter and a 70 mm tip length. The inter-valve distance was related to the leakage resulting from instrument insertion.

(6) *Instrument insertion*: holding the instrument tip between both valves of a 5 mm atraumatic grasper and a 12 mm stapler in the respective trocar sizes.

To verify that the manipulations and insertion test did not cause significant degradation in trocar leak performance, the no manipulation test was repeated after the manipulation and insertion tests.

### Data collection & processing

As the container used for this model was rigid, the inlet flow needed to maintain the pressure equalled the leak through the trocar and/or instrument. The insufflation pressure and trocar leak were measured using a pneumotachograph (Hans Rudolph, series 8410A) combined with differential pressure sensors. These sensors sampled at 200 Hz. Before every experiment the flow measurement was calibrated to room air using a 100 mL syringe (Hans Rudolph 5510 Series). Pressure and leak measurements were recorded using LabVIEW 2019 (National instruments, Austin, Texas, U.S.). For each baseline, manipulation or insertion measurement a separate recording was made. Data processing was performed using Matlab (R2020a, Mathworks, Natic, Massachusetts, U.S.).

The manipulations during the measurements initially caused disturbances in the flow data, after which the flow stabilised to a steady-state leakage. Before visual inspection of the recording, a low-pass filter was applied with a cut-off frequency of 20 Hz. From every recording a sample was visually selected that contained this steady-state leak. In the axial manipulation test, the sample was visually selected to contain 5 oscillations. The minimal sample length for all selected samples was 0.5 s. After selection, samples were averaged.

Since the 12 mm trocars were tested with 5 mm and 12 mm rods and instruments, two baseline measurements were available. Therefore, an additional comparison was made to verify that the manipulations and insertion test did not damage the trocar.

## Results

### Included trocars and instruments

The inquiry for trocars under EAES members resulted in the inclusion of 22 trocars which are listed in Table 1. Regarding the valves inside the trocars, the following observations were made: Most of the 5 mm trocars had 2 valves. Trocars *f* and *k* appeared to have one valve, however after disassembling those two trocars, *f* turned out to have two different valves stacked on top of one another. Trocar *k* had a single component valve in which a diaphragm valve was combined with a cross flap valve, so this was categorized as single valve. Trocar *I* was the only trocar which had three valves having an additional valve after valve 2. Valve 2 in trocar *I* serves the same purpose as in the other trocars. Some of the 12 mm trocars came with a removable diaphragm adapter for use with a 5 mm instrument. In that case, its diameter is shown in the table.

**Table 1:** Properties of the included trocars, each indicated with a letter; lower-case letters indicate a 5 mm trocar, upper-case letters represent the 12 mm trocars

Label	Size (mm)	Use type	No. of valves	Valve 1		Valve 2	Internal valve distance (mm)	Adapter valve diameter (mm)
				Type	Diameter (mm)	Type		
a	5	Reusable	2	Diaphragm	3.5	Flap	30	
b	5	Reusable	2	Diaphragm	2.8	Barrel	30	
c	5	Reusable	2	Diaphragm	3.5	Flap	35	
d	5	Reposable	2	Integrated Diaphragm	4	Bicuspid	15	
e	5	Disposable	2	Diaphragm	2.8	Bicuspid	15	
f	5	Disposable	2	Diaphragm	4	Quadcuspid	0	
g	5	Disposable	2	Diaphragm	2.5	Bicuspid	19	
h	5	Disposable	2	Diaphragm	2.5	Bicuspid	19	
i	5	Disposable	2	Diaphragm	2	Quadcuspid	10	
j	5	Disposable	2	Diaphragm	3.4	Bicuspid	4	
k	5	Disposable	1*	Diaphragm/cross flap	0	NA	NA	
A	12	Reposable	2	Segmented Diaphragm	1.5	Bicuspid	18	
B	12	Disposable	2	Diaphragm	3.5	Bicuspid	15	
C	12	Disposable	2	Diaphragm	3.8	Bicuspid	19	2.4
D	12	Disposable	2	Diaphragm	3.3	Bicuspid	20	
E	12	Disposable	2	Diaphragm	5	Flap	7	2.2
F	12	Disposable	2	Diaphragm	4	Quadcuspid	17	
G	12	Disposable	2	Diaphragm		Bicuspid	32	
H	12	Disposable	2	Diaphragm	3.5	Bicuspid	22	4
I	12	Disposable	3^+^	Segmented diaphragm	0.5	Bicuspid + Diaphragm	16	
J	12	Reusable	2	Diaphragm	9.5	Flap	49	
K	12	Disposable	2	Diaphragm	6.5	Flap	18	

*This valve was a combination of a diaphragm and cross flap valve, it was considered to be a single valve since it was a single component without any distance between the two parts

^+^Behind the bicuspid valve another diaphragm valve was placed. This was considered an extra valve placed directly adjacent to valve 2

The diameters of the first valve ranged from 0–4 mm in the 5 mm category to 0.5–9.5 mm in the 12 mm category. For the first valve the most common (18/22) choice was a diaphragm valve, the other (4/22) were a variation of the diaphragm valve. For the second valve a broader variety of valves was present. The most common choice was a bicuspid valve (11/22), other (9/22) valves used were flap (5); quadcuspid (3); barrel (1) and bicuspid diaphragm (1) type valves. Internal valve distance ranged between 0–35 mm for 5 mm trocars to 7–32 mm for 12 mm trocars.

The consistency of the trocars’ performance was verified after comparing the ‘no manipulation’ results at the start and end of the measurement series. The degradation over a measurement series was found to be less than 0.1 L/min, with the exception of *G*, which was only tested with a 5 mm instrument due to failure during the 12 mm instrument test. The 5 mm results of trocar *G* were still included as the baseline measurement differed by 0.01 L/min. *J* and *K* were only tested with a 12 mm instrument because the first valve diameter was too large for use with a 5 mm instrument.

In total, 26 instruments were tested for leakage: 5 mm disposable instruments (14/26) and 5 mm reusable instruments (6/26). Six instruments had diameters larger than 5 mm: 10 mm (2/26) and 12 mm (4/26), grouped as 10/12 mm instruments. No reusable instruments with a larger diameter were available.

### Baseline leak

**Fig. 3:**
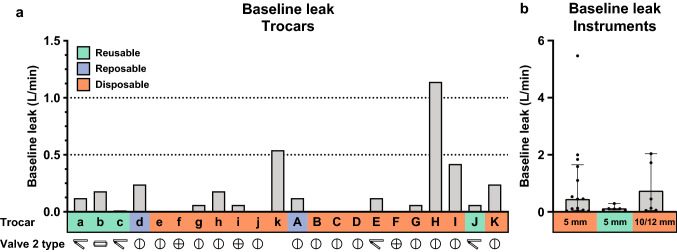
**a** Baseline flow measurements in trocars at 15 mm Hg. Along the *x*-axis the trocar names are noted, the colours correspond to the trocar type. The type of valve 2 is denoted by a symbol under the result of each trocar and is detailed in Table 1. As trocar k had only one valve, it does not have a symbol. **b** Flow through measured instruments. The bar height indicates the median leak for each group, the crosses shows the inter-quartile range and the dots represent the result of each individual instrument

The results of the baseline leak measurements for the trocars and instruments can be seen in Fig. 3. Figure 3a shows the measured leak in the individual trocars. The median leaks were 0.06 L/min with an interquartile range (IQR) of 0 to 0.18 and 0.06 L/min (IQR 0–0.24) for 5 and 12 mm trocars, respectively. In an empty trocar, valve 2 blocks the airflow through the trocar. Therefore, the results of this measurement are related to the performance of valve 2.

Figure 3b shows measured leak through instruments. The results were grouped by instrument size and reusability. The 5 mm disposable instruments had a median of 0.45 L/min (IQR 0.06–1.7), the 5 mm reusables the median was 0.11 (IQR 0.07–0.16), 10/12 mm disposables had a median of 0.29 L/min (IQR 0.06–1.8). This meant that the 5 mm reusable instruments had the lowest median and IQR leak.

### Manipulations

**Fig. 4:**
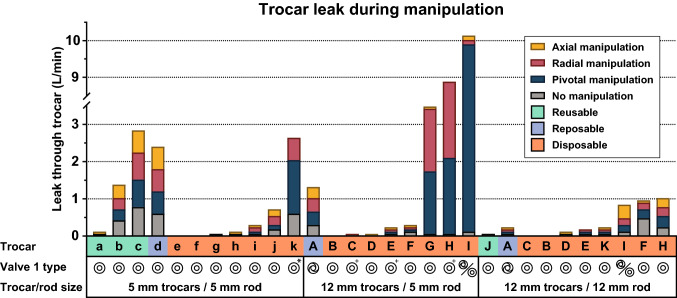
Leak through trocar caused by different manipulations with a solid shaft. The symbols below the bars indicate the type of valve 1 that was used on each trocar. The shaft size, number of valves and adapter valve are indicated below the graph. Trocar I was equipped with a third valve. *Single valve. ^+^Removable diaphragm 5 mm adapter valve

Figure 4 shows the leak through the trocar when the trocar is manipulated with a solid shaft. The results are stacked to show the leak results of each manipulation that is related to valve 1. In the figure we see that there is a large variation in leak between trocars caused by different manipulations. Even within their respective groups, trocars differ in the amount of leakage caused by the individual manipulations.

### Instrument insertion & valve distance

**Fig. 5:**
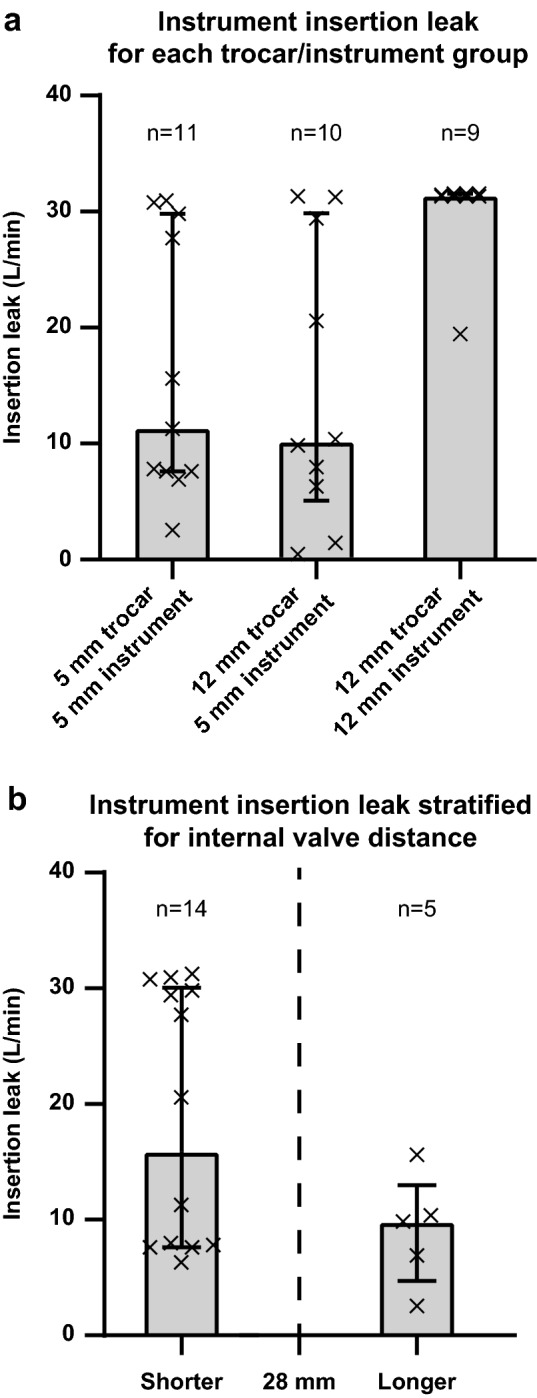
**a** Leak during instrument insertion trocars grouped per trocar size and used instrument size, median and inter-quartile range per group. **b** The effect of valve distance compared to the tool tip length of the surgical instrument, in this case longer or shorter than 28 mm. The height of the boxes is the median value, the whiskers represent the inter-quartile range and the crosses are each individual trocar

In Fig. 5a the flow through the trocars during instrument insertion can be seen. Both the 5 mm and 12 mm trocars achieved varying results when tested with the 5 mm instrument. The 5 mm trocars with 5 mm instrument had a median of 11.3 L/min (IQR of 7.6–29.8), the 12 mm trocar with 5 mm instrument had a median of 10.1 L/min (IQR of 5.1–29.9), the 12 mm trocars with 12 mm instrument had a median of 31.3 L/min (IQR 31.3–31.6). In the 12 mm trocars with 12 mm instrument group, the measurement results do not reflect the actual leak as it was outside the saturation limit of the sensor.

Figure 5b shows a plot of the instrument insertion test, in which all trocars are divided into two groups: trocars with an inter-valve distance larger and shorter than the instrument tip. The figure shows that the trocars with a larger inter-valve distance had a smaller variation in leakage, than trocars with a smaller distance between the valves.

## Discussion

The results of this study show the potential of gas leakage pathways through laparoscopic trocars and instruments. A wide range of leakages through trocars and instruments was found under varying conditions by utilising a protocol with different interactions. These findings show that the choice of equipment as well as the circumstances under which the equipment is used determine the exposure level of OR personnel.

### Interpretation of results

#### Baseline

In trocars, valve 2 prevents gas from escaping the peritoneum. Therefore, the results of the baseline measurement are related to the properties of this valve. However, no clear relation was found between valve type and performance which becomes apparent when observing the large variance in performance of the most used valve: the bicuspid valve.

The median baseline leak of instruments is higher than that of trocars. The results do show a great variation within comparable instrument types. For example, a tenfold difference in leakage was measured between two 5 mm tissue sealing devices of different brands. The choice of instrument type and reusability has a large influence on the total gas leakage, which becomes apparent when comparing the medians and IQR of the 5 mm instrument types. Between these categories, reusable 5 mm instruments perform better than the disposable 5 mm instruments. Upon inspection, the reusable 5 mm instruments were fitted with a rubber seal at the proximal end. Testing the effect of removing this seal could not be tested, yet this seal is expected to have prevented a large portion of the gas flow through the instrument. It is unclear why other manufacturers have not included similar measures for their disposable instruments.

#### Manipulation

Trocars that perform well during baseline measurements, do not always perform well during manipulation. During the largest portion of a surgical procedure, trocars will be manipulated by an inserted instrument. Therefore, the performance of trocars during surgical manipulation will significantly determine the overall performance in gas leakage of the trocars. The results in Fig. 4 show the rate of leakage during each manipulation, which does not represent leakage during surgery.

There are several additional factors that need further research before the results can be used to predict actual leakage during surgery. Firstly, the frequency and duration of the manipulations during surgery is unknown. These are needed to determine the ratio at which the leakage during each respective manipulation occurs.

Secondly, the manipulations in this study were performed to their maximum effect. For instance, the pivotal manipulation was performed with external stabilisation such that the instrument insertion leakage was reached. In reality, the pivot angle of the trocar is limited by the compliance of the abdominal wall. Therefore, leakage caused by pivoting the trocar will be less during surgery than in this study and will depend on the mechanical properties of the trocar valves and the patient.

Lastly, the steel rods used in this study were selected to match the marketed standard diameters of 5 and 12 mm instruments. In reality, these dimensions vary and could result in higher or lower leakages depending on the interaction between the trocar and instrument.

From the measurements it seems that the 12 mm trocars with 5 mm instruments maintain a less reliable seal when compared to 5 mm instruments in 5 mm ports, especially during pivotal and radial manipulation. Some 12 mm trocars are more successful in accommodating smaller size instruments than others. Several trocars have measures for this, such as trocar *C, E*, and *H*, yet the apparent measures, such as an adapting valve, do not guarantee a good seal, as can be seen in Fig. 4. The additional valve in trocar *I*, does also not increase the performance under manipulations.

#### Insertion

As seen in Fig. 5, many of the trocars were susceptible to leaks during instrument insertion, especially in trocars that have a small inter-valve distance. The tip length of the 12 mm instrument was much larger than the inter-valve distance of the 12 mm trocars. Despite reaching the sensor saturation limit, Fig. 5a shows that none of the 12 mm trocars were able to successfully prevent leakage caused by the 12 mm instrument.

### Limitations

A total of 11 5-mm trocars were tested and 12 12-mm and 5/12-mm trocars were tested. Each trocar was tested once. The authors were aware that individual trocars might not always be representative for a larger sample. Even after careful inspection of trocars, defects might have gone unnoticed.

Not all trocar and instrument manufacturers were represented in this study. The authors were limited to the equipment that was provided, which could therefore be a source of bias. Because of the large variation in trocar valve types and geometries, it was not possible to directly show a statistical relation with the leakage results. For example, we cannot make claims of the performance of reusable over disposable trocars.

This study did not investigate the incision leak pathway and had only one sample of most trocars and instruments available. The results of this study should therefore not be considered as a recommendation of specific trocars, but should provide information on the leak performance in relation to specific design properties.

### Contamination and leakages

The work by Stotz et al. [1] describes the type and usage of instruments and trocars during a median of 103 gynaecological laparoscopic interventions. This data can be used to estimate leakage during a hypothetical intervention to add perspective to the individual measurement results of this study.

During the interventions, a four-trocar arrangement was used: one 12 mm trocar for the endoscope, two 5 mm trocars for 5 mm surgical instruments, and one 12 mm trocar used for 12 mm surgical instruments. On average 20 instrument changes take place per hour per trocar. The results of the instruments and trocars were used to extrapolate this data. The 25th and 75th percentiles are taken to indicate the spread in leak that was observed.

During surgery, the trocars had no instruments inserted for 16.4 min per hour. This situation was measured during the trocar baseline measurements and results in leakages of 0.2 L/hr and 8.8 L/hr for the 25% worst and 25% best performing trocars.

For 43.6 min per hour, an instrument was inserted into a trocar. When an instrument is inserted, gas escapes past the trocar valves and through the instrument. These were measured during the instrument baseline measurement, which showed that leakage through the instruments contributes most to the total leakage. Because more data is needed to incorporate each manipulation, only the ‘no manipulation’ condition is included in the calculation. Combining the leakage of the 25% best and 25% worst performing trocars (8.5 L/hr and 54.4 L/hr) and instruments (8.3 L/hr and 79.4 L/hr), results in 16.9 L/hr and 133.8 L/hr, respectively.

Each of the twenty times per hour the instruments are switched, there is an increase in leakage that was assumed to last one second. The level of increase was measured and presented in Fig. 5. For the two 5 mm and one 12 mm working trocars, the instrument switches contribute 15.5 L/hr and 29.7 L/hr for the 25% best and 25% worst performing trocars.

In total, the choice of equipment can result in a difference of 140 L/hr of escaping CO_2_. This example shows that a large variation can be expected between different combinations of trocars and instruments. The impact of this leakage on surgical safety is part of the ongoing debate on the risks of contaminated gas and air in the surgical environment.

Although large volumes of gas seem to have a large influence on the exposure of OR staff to insufflation gas, the type of leakage is equally important. When inserting an instrument a small burst of gas leaks from the trocar. This higher speed might have more severe consequences for the safety of operating theatre personnel as it is released directly into the surgical workspace.

The OR ventilation system will have a major influence on how long gas remains present in the surgical workspace. Operating room airflows are difficult to predict because of the dynamic nature of this workplace. Limiting CO_2_ leakage at the source of the equipment, by means of a redesign, could be an alternative way to minimise exposure. The results of this study stress the importance of leak performance indicators for careful equipment selection.

### Recommendations

For healthcare personnel concerned about gas leakage, there is no method for choosing equipment based on leakage requirements, as manufacturers do not readily provide this information. Currently, there is no universal leakage testing standard for trocars and instruments, such a standard would allow comparison of leakage performance between manufacturers. Additionally, a standardized testing method could also detect equipment failures in reusable trocars after routine maintenance. Wear and damage to the valves during sterile reprocessing of equipment is easily overlooked. For example, two reusable trocars were excluded from the results because they showed much higher leakage during baseline testing. After close inspection, we discovered that the seals under the flap valve were torn or missing. Investigation of wear over time requires comparison of leak performance between multiple trocars of an identical brand and type to exclude the effect of individual samples.

Levels of exposure can be minimised in many ways. One safety measure to prevent insertion leakage that can easily be applied, is to pair instruments and trocars based on tip length and inter-valve distance. However, none of the 12 mm trocars had sufficient inter-valve distance to prevent leakage during instrument insertion. An adapted design with a large valve distance would be an option that substantially increases the size of the trocar. An alternative could be an adapter specifically for use with large-tip instruments such as surgical staplers.

The experiments showed that none of the trocars were able to prevent leakage in all of the tests. More research is needed into the influence of aspects such as: valve material, valve compliance or thickness, valve diameters and manufacturing methods. This can be used in the design process of trocars with improved gas leak performance.

## Conclusion

This study quantified gas leaks of equipment in situations related to laparoscopic surgical settings. Not only the individual contribution of trocars and instruments was measured, also the effect of specific trocar-instrument interactions was studied. The results show a large variation between trocars of the same size and type. Additionally, large differences were observed between instruments of different types meant for the same functionality.

Peritoneal gas possibly carries harmful substances into the OR through the identified leakage pathways. For surgical teams willing to select equipment based on their leak performance, it is difficult to make the selection based on geometric properties and appearance. Therefore, manufacturers should standardise reporting on the leakage performance and incorporate leakage in the design process of laparoscopic equipment.
