# Correction to: Characterisation of trocar associated gas leaks during laparoscopic surgery

**DOI:** 10.1007/s00464-021-08897-x

**Published:** 2021-11-23

**Authors:** Daniel Robertson, Frank Sterke, Willem van Weteringen, Alberto Arezzo, Yoav Mintz, Felix Nickel, the Technology committee of the European Association for Endoscopic Surgery (EAES), Luigi Boni, Ludovica Baldari, Thomas Carus, Manish Chand, Hans Fuchs, Fanny Ficuciello, Stefania Marconi, George Mylonas, Young Woo Kim, Kiyokazu Nakajima, Marlies Schijven, Pietro Valdastri, Chen Sagiv, Pietro Mascagni, Piotr Myśliwiec, Wanda Petz, Francisco Sánchez-Margallo, Tim Horeman

**Affiliations:** 1 Department of Biomechanical Engineering, Faculty of Mechanical Engineering, Delft University of Technology, TU Delft, Mekelweg 2, 2628 CD Delft, The Netherlands; 2 Department of Paediatric Surgery, Erasmus MC Sophia Children’s Hospital, University Medical Center Rotterdam, Rotterdam, The Netherlands; 3 Department of Surgical Sciences, University of Torino, Torino, Italy; 4 Department of General Surgery, Hadassah Hebrew University Medical Center, Jerusalem, Israel; 5 Faculty of Medicine, Hebrew University of Jerusalem, Jerusalem, Israel; 6 Department of General, Visceral, and Transplantation Surgery, Heidelberg University Hospital, Im, Neuenheimer Feld 420, 69120 Heidelberg, Germany

## Correction to: Surgical Endoscopy 10.1007/s00464-021-08807-1

This article was updated to correct the order of the author listing.

